# Supplementary figures and images for: Functional-genomic analysis reveals intraspecies diversification of antiviral receptor transporter proteins in Xenopus laevis
Source: PLoS Genet. 2021 May 20;17(5):e1009578. doi: 10.1371/journal.pgen.1009578 (PMC8172065; doi:10.1371/journal.pgen.1009578)

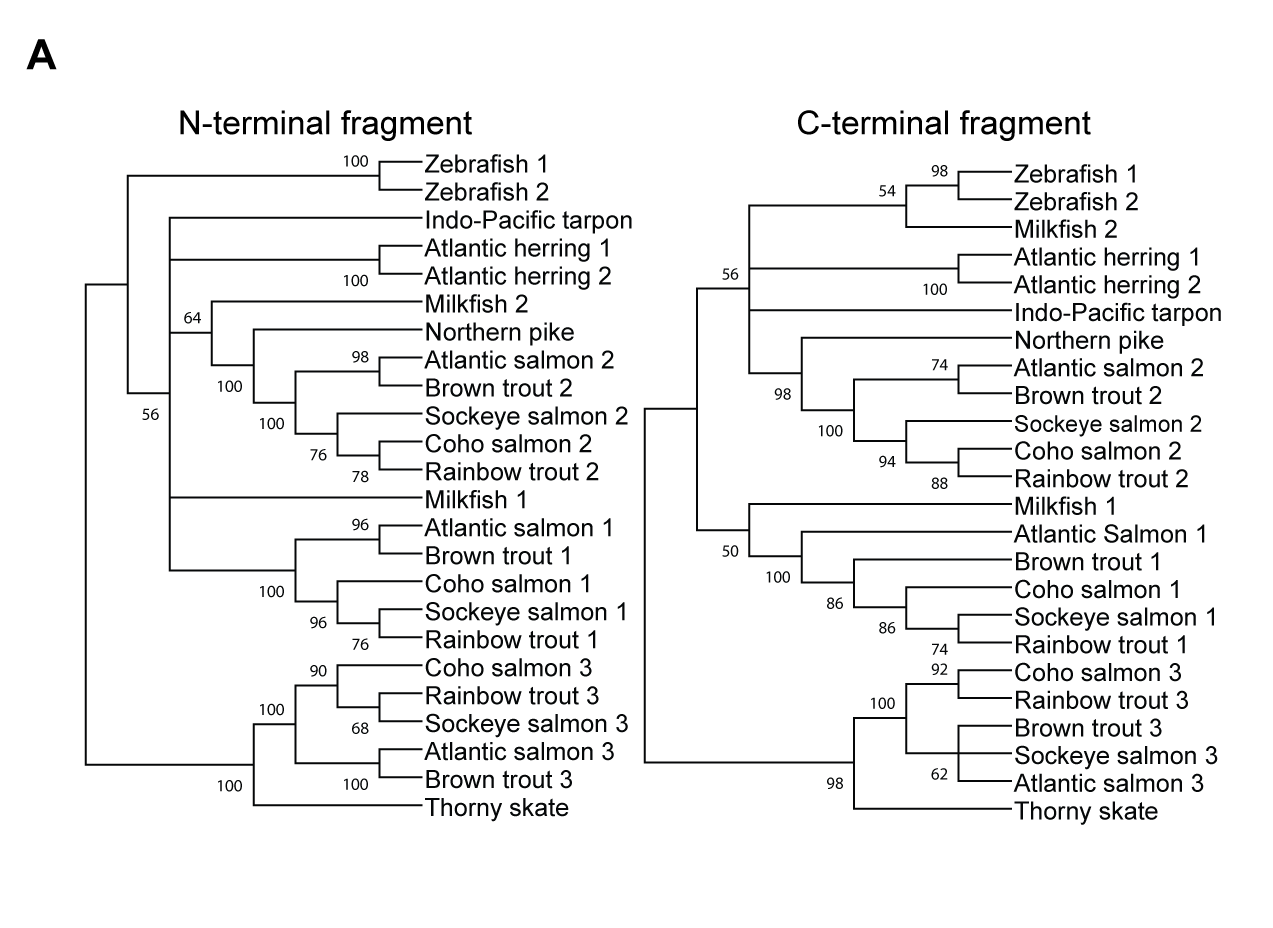

Supplement: S1 Fig — A)Collapsed maximum-likelihood trees for N-terminal and C-terminal fish RTP gene fragments, relative to a predicted recombination breakpoint (nt383). Numbers indicate the percentage of 50 bootstrap replicates for which the associated taxa clustered together. Nodes with <50% bootstrap confidence were collapsed. Trees were generated as in Fig 1B. (TIF) [file pgen.1009578.s001.tif]

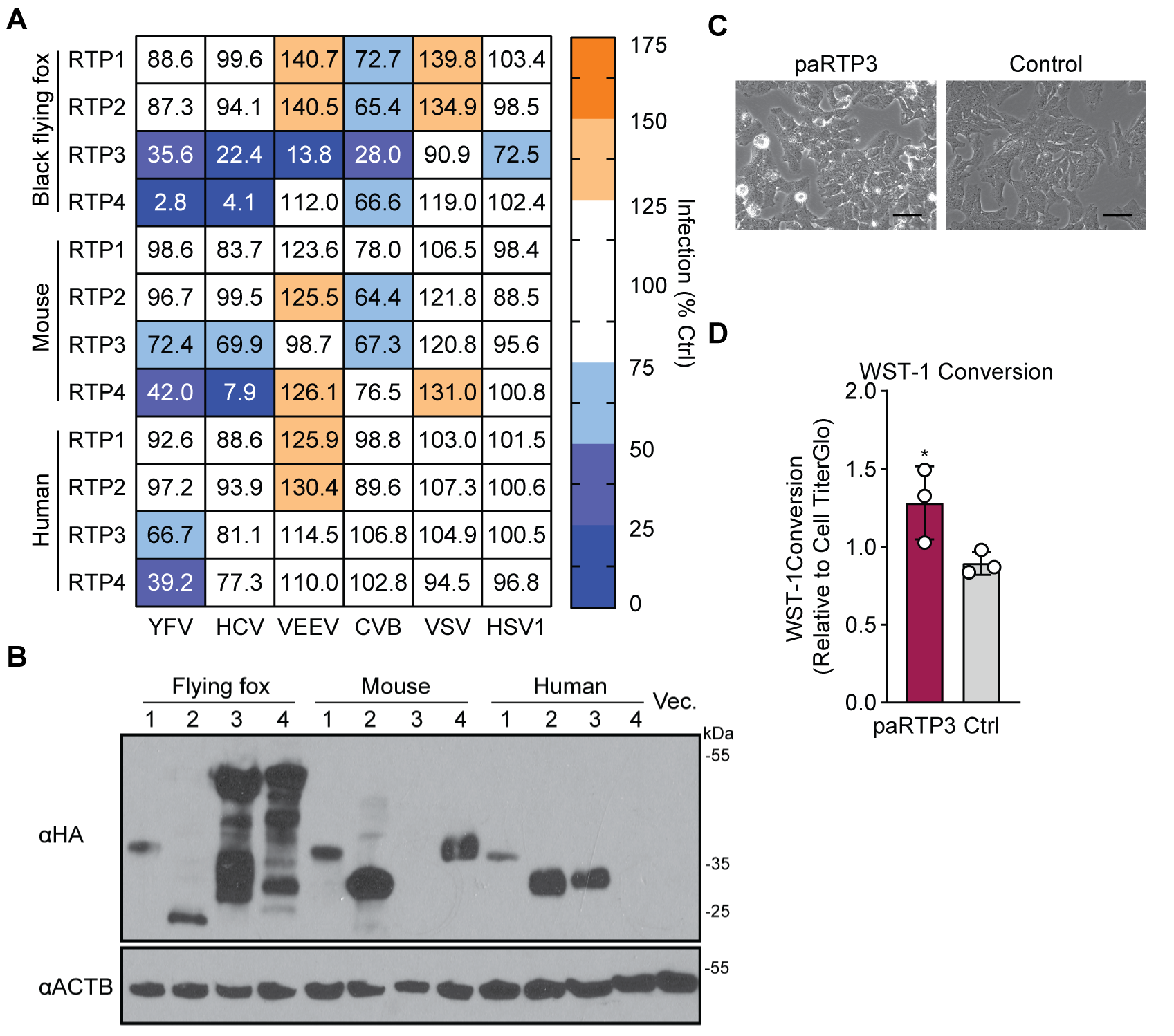

Supplement: S2 Fig — A) Huh7.5 cells ectopically expressing the indicate RTPs or a vector control were infected with a panel of viruses at an MOI of 0.5 to 1. Cells were harvested after the completion of approximately one replication cycle and percent infection was assessed by flow cytometry. Cells represent the mean of n = 2 (HCV) or n = 3 (all other viruses) biological replicates, normalized to control. B) Western blot of ectopically-expressed RTPs. For each screen replicate, an additional well of cells was harvested alongside infections for protein expression analysis. Blot is representative of n = 3 replicates. C) Representative micrograph of paRTP3-expressing Huh7.5 cells and vector control cells. Scale bar: 20μm. D) Huh7.5 cells expressing the indicated construct were seeded at 10,000 cells per well on a 96-well plate one day prior to assay. The day of WST assay, WST reagent (Takara) was added (10uL in 100uL media) and cells were incubated for two hours, after which visual absorbance was assayed. In parallel, cell density was measured using Cell Titer-Glo (Promega) and luminometry. (TIF) [file pgen.1009578.s002.tif]

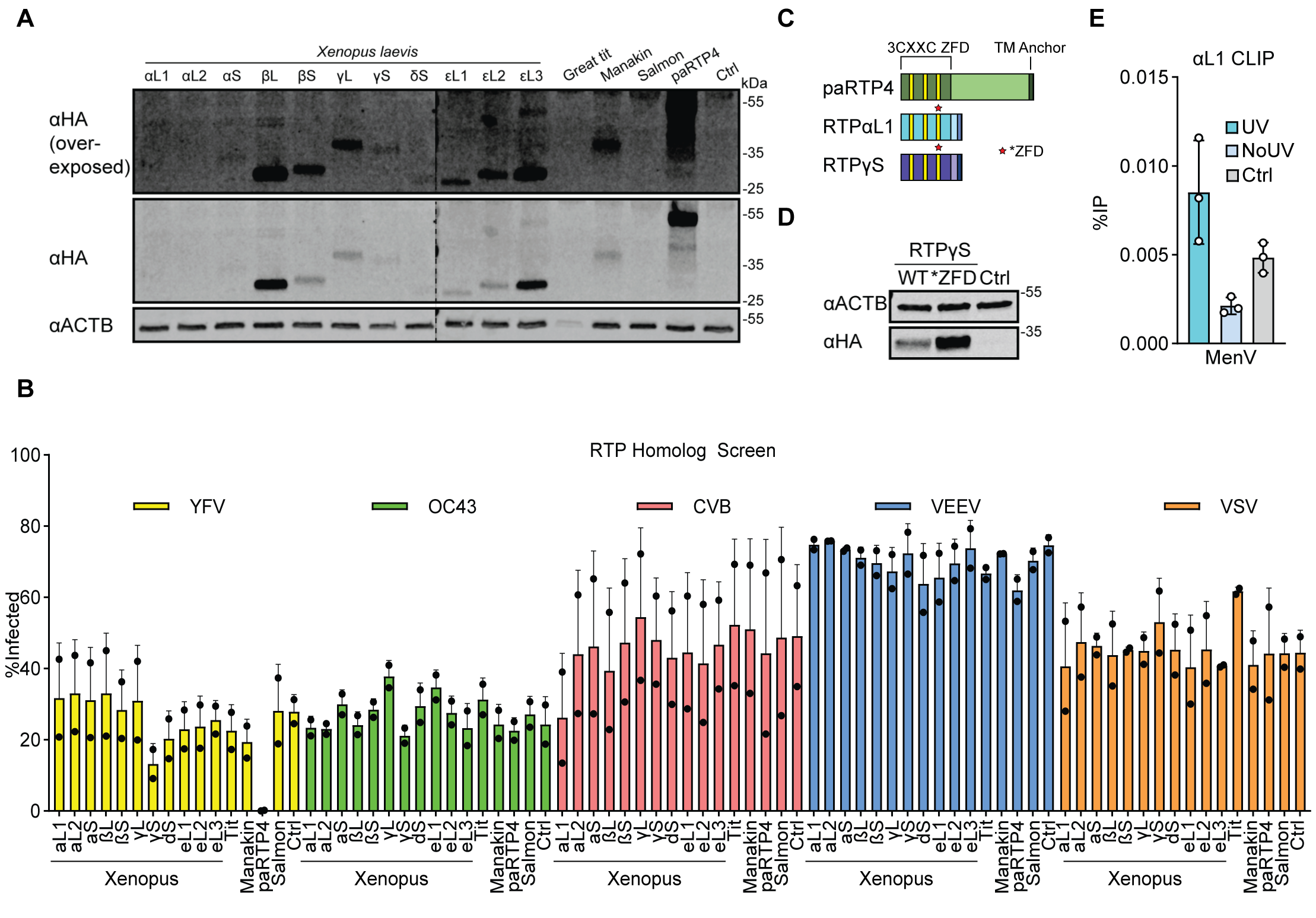

Supplement: S3 Fig — A) Western blot of ectopically-expressed RTPs. Vertical dotted line indicates splice site for two independently-run gels. For each screen replicate, an additional well of cells was harvested alongside infections for protein expression analysis. Blot is representative of n = 2 replicates. B) Raw data related to Fig 4A. Huh7.5 cells ectopically expressing the indicate RTPs or a vector control were infected with a panel of RNA viruses at an MOI of 0.5 to 1. Cells were harvested after the completion of approximately one viral life cycle and percent infection was assessed by flow cytometry. Bars represent the mean ± SD of n = 2 biological replicates. C) Cartoon representation of X. laevis RTPαL1 and RTPγS with paRTP4 as a reference. Yellow boxes denote N-terminal CXXC motifs, the third of which was targeted for disruption by site-directed mutagenesis to generate *ZFD RTPs. D) Western blot of ectopically-expressed RTPγS and RTPγS*ZFD. Blot is representative of n = 2 replicates. E) Huh7.5 cells expressing HA.RTPαL1 or a vector control were infected with MenV (MOI of 2) for 6h. CLIP-qPCR identified RNA bound by HA.RTPαL1. UV: UV crosslinked HA.RTPαL1 cells. NoUV: non-crosslinked HA.RTPαL1 cells. Vector: UV crosslinked vector control cells. Bars: mean ± SD of n = 3 biological replicates. (TIF) [file pgen.1009578.s003.tif]
