# Supplementary material for: Functional-genomic analysis reveals intraspecies diversification of antiviral receptor transporter proteins in Xenopus laevis
Source: PLoS Genet. 2021 May 20;17(5):e1009578. doi: 10.1371/journal.pgen.1009578 (PMC8172065; doi:10.1371/journal.pgen.1009578)
Supplement: Attachment — Submitted filename: Response to Reviewers.docx [file pgen.1009578.s010.docx]

Reviewer #1: PGENETICS-D-21-00228

This manuscript describes the evolutionary comparison of receptor transporter proteins (RTPs) and focuses on the diverged and expanded evolution of these in X. laevis. The authors underline the expansion of the Xenopus RTPs, show that RTPs from several species are upregulated in response to immune challenge and that some of them are important during RNA virus infections.

Please find my comments and suggestions below.

In your introduction, please elaborate on the mechanism by which RTP4 restricts viral replication.

We thank the reviewer for their suggestion and have included additional discussion of the antiviral mechanism of RTP4 in the introduction [Lines 41-44 in the revised manuscript].

Fig. 1 is very difficult to read. Please consider increasing the resolution in A and B and increasing the font or somehow highlighting key species in A.

We appreciate the reviewer bringing our attention to the cluttered nature of Figure 1, particularly panel 1A. We have made several modifications to improve readability, including increased font size across all panels. We additionally decided to simplify panel 1A by removing species names, since its primary point is to demonstrate the divergence of groups of RTPs, not of individual species. We now include the corresponding Newick file in Supplemental File 1 so that a reader can look at the relationships between RTPs from specific species, if so desired.

The synteny analyses in Fig. 2A is difficult to discern. Please clearly indicate the respective RTP genes including numbers and label several of the neighboring genes. Please also indicate the s and L allelic forms of these genes in X. laevis.

Per the reviewer’s suggestion, we have added additional neighboring genes to the synteny analysis in panel 2A. We have also made efforts to improve the visual representation of the data by increasing font size and by making chromosomes and allelic forms of *X. laevis* genes more clear. We also now label all RTPs species to match the labeling used throughout the rest of the manuscript.

There is a gap between pg. 6 and 7. Possibly formatting?

We appreciate the reviewer’s attention to detail. This gap results from our inclusion of Table 1 in-line, per journal guidelines. We opted to include a page break above the table to avoid wrapping the table across a page.

Reviewer #2: In this manuscript, the authors perform an evolutionary analysis of Receptor Transporter Proteins (RTPs) in vertebrates. RTP4 in humans is an interferon stimulated gene and was previously shown by this group to have anti-flavivirus activity. Here, they identified signs of positive selection of RTPs in several species, which often indicates virus-host conflicts driving accelerated evolution. They further identified expansions of RTPs in certain species, including xenopus laevis, which encodes 11 RTPs. They went on to provide the first RNA seq timecourse of xenopus cells stimulated with the viral mimic polyI:C. This is a valuable dataset in and of itself, and this identified xenopus RTPs that were upregulated by polyI:C stimulation. They then ectopically expressed several xenopus RTPs in human cells and discovered antiviral activities against specific viruses or virus families, including flaviviruses and picornaviruses. These data provide some of the first evidence that antiviral activity of RTPs is present outside of mammals and is thus evolutionarily more ancient than previously appreciated. Overall, I find this paper to reach the threshold of both significance and quality for publication in PLOS Genetics with only minor text additions as noted below.

1. While the authors appropriately mention certain limitations of their study, one limitation that was not mentioned was the lack of infection or RTP manipulation in xenopus cells. If this is technically challenging, a brief discussion of this would be helpful.

We agree with the reviewer that this is an additional limitation of our study, and now discuss this as an important future direction in our manuscript [Lines 266-271 in the revised manuscript]. We additionally detail our attempts to perform infections and manipulations in Xenopus cells below:

We found that X. laevis A6 cells are slightly permissive to certain flaviviruses, including yellow fever virus. However, a combination of low permissivity (high MOI infections do not lead to infection of all cells), cell-intrinsic immunity (pharmacological inhibition of TBK1 modestly enhances infection), and cell death (infected cells die and infection does not efficiently spread) reduce the practicality of A6 cells as a model for YFV infection (Response Figure 1). We obtained similar results with Zika virus and Entebbe bat virus.

Response Figure 1. Left: Fluorescence imaging of A6 cells infected with YFV-Venus at an MOI of 1 following two-hour preincubation with Compound II (PMID 21329883) or DMSO. In the absence of TBK1 blockade, few cells infect and those which do rapidly undergo cell death. With blockade, infection spreads but cells still ultimately undergo cell death. Fields are representative and do not necessarily represent the same set of cells across tie points. Image is pseudocolored green to match GFP fluorescence. Right: A6 cells were infected at an MOI of 70 with YFV-17D and stained for double-stranded RNA (viral replication intermediate) 24 hours post-infection.

Regarding transgene expression efficiency in A6 cells, we confirmed the results of the paper we now cite in our discussion (PMID 21959846). While we were able to transfect A6 cells with RFP-expressing plasmids at a low efficiency, most cells die following transfection. Reduction of plasmid dose did not seem to reduce cell death, instead only decreasing efficiency (Response Figure 2). Additionally, lentiviral transduction and AAV-based gene delivery methods were unsuccessful in our hands (not shown).

Response Figure 2. A6 cells (150,000 cells/well on a 12w plate) were transfected with RFP-expressing plasmid (pSCRPSY-EMPTY) using Lipofectamine 3000 (Invitrogen).

2. It would be nice if the authors could present the data in the supplementary tables in a more user-friendly way with captions and table headings. For example, in Supp Table 2, a simple list of the upregulated xenopus gene names and fold upregulation at each timepoint would make the dataset more accessible for quick inspection by the average reader who may want to quickly scan for a specific ISG.

We thank the reviewer for their suggestions for how we can enhance the utility of our dataset for the community and have made efforts to do so. Regretfully, the Xenopus laevis genome annotation lags behind those of other model species, and many transcripts are not assigned gene names. We have made efforts to better annotate many of the transcripts, and additionally have made formatting changes to improve readability. Per the reviewer’s suggestion, we now include a list of top upregulated Xenopus transcripts, along with fold upregulation at each time point, as the first tab in Supplemental Table 2. We have additionally included more headings and notes in this and other supplemental tables.

Reviewer #3: This is a very thorough and well performed study where authors describe the immunogenetics of antiviral RTPs in amphibians. The team reports an expansion of these molecules in Xenopus (11 RTPs) and perform in-depth phylogenetic analyses to ascertain the evolutionary history of RTPs in amniotes and anamniotes. They additionally analyze differences between the Xenopus S and L genomes. The authors also present a series of in vitro assays using an ectopic expression system to show responses to flaviviruses and in vitro transfection of Xenopus A6 cells with poly I:C. I enjoyed the CLIP experiments that point towards similar antiviral mechanisms of Xenopus RPL and mammalian RPL4.
The manuscript is well written and the experiments include the correct controls.

I have two main questions:

1- Where are RTPs expressed in X. laevis (what tissues) at the steady state? Are they constitutively expressed? Is there high expression in the olfactory system as mentioned at the end of the discussion compared to lymphoid tissues?

We thank the reviewer for this suggestion. We assessed RTP expression levels using data available at Xenbase and found intriguing expression patterns that we now address in our discussion [lines 272-284 in the revised manuscript].

2.- Authors state the main limitation of this study in lines 252-255 of the discussion. Given all the published RNA-Seq datasets of amphibians infected with amphibian-relevant pathogens such as ranavirus and Bd, I highly recommend that the authors complement their in vitro findings with in vivo data that has already been published. Data mining these datasets will help support the idea that amphibian RLPs are indeed antiviral in a more biologically relevant model. Further, having the comparison between Bd versus ranavirus datasets would be really interesting to ascertain antiviral specific roles.

We appreciate the reviewer’s recommendation. We are hesitant to connect expression in response to a specific pathogen and antiviral function in the absence of experimental support, as we expect that any virus that elicits a PAMP-driven antiviral response would prompt the induction of RTPs that were upregulated following poly(I:C) stimulation. However, we agree that demonstrating such upregulation *in vivo* as part of an antiviral response would be valuable. Unfortunately, outside of one example (detailed below) we have encountered difficulty in finding datasets that include RTPs. In particular, most amphibian infection datasets involve *de novo* transcriptome assembly and RTPs are not included in the available processed data. Further, the lack of reference genomes for these *de novo* transcriptome assemblies precludes our ability to look for mapped reads at the RTP locus in genome browsers. Re-analyzing such RNA-seq data for RTP induction would indeed be valuable, perhaps once reference-quality genomes for more amphibian species are available. We provide a summary of our efforts to mine available datasets below:

- Bd, X. laevis: PMID 19701481 – RTPs are not included in the microarray
- Bd and Bsal fungal coinfection, N. viridescens: PMID 32310322 – *de novo* transcriptome assembly. One RTP, called “RTP3” is shown to be significantly upregulated in infected newts. We now cite this paper as evidence of RTP induction as part of an immune response in amphibians [lines 161-163 of the revised manuscript]. We, however, hesitate to speculate that this is suggestive of a role for RTPs in fungal immunity since many immune genes with no specific activity towards a given pathogen will be induced by a variety of stimuli. There are no paired Ranavirus datasets for more in-depth comparisons.
- Ranavirus and Bd, R. temporaria: PMID 26111016 – RTPs are not included in the *de novo* transcriptome assembly
- Ranavirus, A. davidianus: PMID 29558886 – *de novo* transcriptome assembly. Outside of the top ten upregulated genes, reported DEGs were filtered for those with a known immune function, which was not appreciated for RTPs at the time of publication.
- Bd, L. verreauxii: PMID 29509187 – *de novo* transcriptome assembly. Extensive annotation of transcripts available but none are called as RTPs.

Minor:
- RNA-Seq data: I cannot find the number of replicates per treatment group in the materials and methods

We have clarified in the materials and methods that this experiment was performed in biological triplicate [lines 346-347 in the revised manuscript].

- Phylogenetic trees: I cannot find the accession numbers of the sequences for all the RTP molecules used in Figure 1.

We now include a table of accession numbers for RTP sequences as Supplemental Table 4.

**Have all data underlying the figures and results presented in the manuscript been provided?**
Large-scale datasets should be made available via a public repository as described in the *PLOS Genetics* [data availability policy](http://journals.plos.org/plosgenetics/s/data-availability), and numerical data that underlies graphs or summary statistics should be provided in spreadsheet form as supporting information.

Reviewer #1: Yes

Reviewer #2: **No:** I don't think I saw all of the underlying data for bar graphs present in any of the supplementary tables, though I'm not sure this is necessary since their bar graphs include overlay of individual data points.

We thank the reviewer for their attentiveness and have compiled a table of all underlying numerical data which we now include as Supplemental Table 5.

Reviewer #3: Yes

PLOS authors have the option to publish the peer review history of their article ([what does this mean?](https://journals.plos.org/plosgenetics/s/editorial-and-peer-review-process#loc-peer-review-history)). If published, this will include your full peer review and any attached files.

If you choose “no”, your identity will remain anonymous but your review may still be made public.

**Do you want your identity to be public for this peer review?** For information about this choice, including consent withdrawal, please see our [Privacy Policy](https://www.plos.org/privacy-policy).

Reviewer #1: No

Reviewer #2: **Yes:** Jacob Yount

Reviewer #3: **Yes:** Dr. Irene Salinas
